# High levels of burnout among health professionals treating COVID-19 patients in two Nile basin countries with limited resources

**DOI:** 10.1038/s41598-023-33399-2

**Published:** 2023-04-20

**Authors:** Noha Ahmed EL Dabbah, Yasir Ahmed Mohammed Elhadi

**Affiliations:** 1 Department of Health Administration and Behavioral Sciences High Institute of Public Health, Alexandria University, Alexandria, Egypt; 2 Department of Public Health, Medical Research Office, Sudanese Medical Research Association, Khartoum, Sudan

**Keywords:** Occupational health, Public health, Human behaviour

## Abstract

Burnout syndrome is a real cause for concern in African health facilities. Healthcare professionals (HCPs) burnout is considered a great public health problem and especially during pandemics as several physical and emotional stressors on this population can lead to increased burnout. This study aimed to investigate the frequency and associated factors of occupational burnout among HCPs working at COVID-19 isolation facilities in Egypt and Sudan. This is important to summarize lessons learned and inform relevant interventions for future pandemic responses. A cross-sectional survey was conducted among frontline HCPs from May 2021 to July 2021. An online, anonymous, self-administered questionnaire was used for data collection. Occupational burnout was estimated using  the Oldenburg Burnout Inventory. A total of 362 HCPs participated in the study and were equally recruited from Egypt (181) and Sudan (181) with a mean age of (31.84 ± 8.32) years. More than half of HCPs were females (60%) and physicians (58.3%). Most HCPs included in the study had high levels of work disengagement (75.4%) and emotional exhaustion (98.6%). Burnout syndrome was present in 75% of the HCPs with 77% among Egyptian HCPs and 71% among Sudanese HCPs. Multivariate logistic regression was used to determine predictors of burnout, working hours per week were the parameters associated with burnout syndrome among Egyptian HCPs; while for Sudanese HCPs, these were age and number of days off. The study revealed a high level of burnout syndrome among HCPs working at COVID-19 isolation facilities in both Egypt and Sudan. Appropriate actions should be taken to preserve the mental health status of HCPs through the establishment of effective and efficient coping strategies.

## Introduction

The coronavirus disease (COVID-19) has threatened global health and sustainable development, it was declared a global pandemic by the World Health Organization in March 2020 and the number of confirmed cases and deaths has been escalating since that time until the virus reached almost all countries^1,2^. The global pandemic has posed unprecedented challenges to health systems worldwide, leading to a considerable impact on healthcare delivery, including the cessation of routine services, service rationings, repurposing of clinical areas, and assigning healthcare professionals (HCPs) to new tasks within unfamiliar high-risk clinical environments^3,4^.

The COVID-19 pandemic has created unique challenges for organizations and individuals, contributing to increased levels of burnout. Organizational causes of burnout during the pandemic may include factors such as high workload due to increased demand for services, lack of control due to rapidly changing guidelines and protocols, insufficient resources such as personal protective equipment, and poor social support due to physical distancing measures. Additionally, the pandemic has created uncertainty and financial strain, leading to a lack of recognition and appreciation for healthcare workers. Individual causes of burnout during the pandemic may include increased stress and anxiety related to fear of contracting the virus, difficulty balancing work and personal life due to changes in routine, inadequate coping mechanisms due to limited access to social support, and personal life stressors such as financial strain and family health concerns. These stressors, combined with the prolonged and unpredictable nature of the pandemic, can lead to emotional exhaustion, depersonalization, and reduced personal accomplishment, which are the hallmarks of burnout syndrome^5–8^.

Recent burnout concept models emphasize that burnout covers the two main core symptoms of high levels of exhaustion and depersonalization because these symptoms are considered to be the most salient features of burnout. Exhaustion refers to a state of physical, emotional, and mental fatigue that results from prolonged stress, while depersonalization refers to a sense of detachment and cynicism towards others, particularly those who are being served or helped. These two symptoms are thought to be closely related, with exhaustion leading to emotional detachment and depersonalization. Reduced accomplishment is often seen as a complication of exhaustion rather than a cause, as it can result from feeling overwhelmed and unable to meet the demands of work. Focusing on the core symptoms of exhaustion and depersonalization allows for a clearer understanding of the underlying mechanisms that contribute to burnout and can help to inform targeted interventions to prevent and treat burnout^9,10^.

The consequences of burnout syndrome during the COVID-19 pandemic can be significant and far-reaching. Healthcare workers suffering burnout may experience physical and mental health problems, such as fatigue, insomnia, anxiety, depression, and burnout-related illnesses such as cardiovascular disease, gastrointestinal problems, and musculoskeletal disorders. Burnout can also lead to decreased job satisfaction, reduced work performance, and increased absenteeism and turnover. Additionally, burnout can have a negative impact on patient care, as burnt-out healthcare workers may be less empathetic, less attentive to patient needs, and more prone to medical errors^11,12^.

There has been a significant increase in levels of burnout syndrome among HCPs during the COVID-19 outbreak^13^. For instance, a global survey of burnout syndrome during the COVID-19 pandemic among HCPs from 60 countries showed that more than half (51%) of HCPs experienced burnout^14^. In the Middle East, the prevalence of occupational burnout among HCPs was reported to be between 40 and 60%^15^. It is essential to study burnout in low- and middle-income countries (LMICs) because these regions often have unique challenges and circumstances that can impact the prevalence and extent of burnout among HCPs. Egypt and Sudan, like many African countries, have limited resources, high workloads, and inadequate staffing, leading to increased stress and pressure on healthcare workers. Additionally, cultural and social factors may influence the experience and expression of burnout in these regions particularly during health emergencies^16^. Understanding the prevalence and impact of burnout in LMICs is crucial for promoting the well-being of healthcare workers and maintaining high-quality patient care. Hence this study aimed to investigate the frequency and associated factors of burnout syndrome among HCPs specifically at the COVID-19 isolation facilities in Egypt and Sudan. Our hypothesis that we wanted to prove is that first, there is a high prevalence of burnout syndrome among front-line HCPs during pandemics such as the COVID-19 outbreak in both African countries, and second we hypothesize that there are certain socio demographic and specific professional characteristics that predict the occurrence of burnout among these HCPs. This is important to summarize lessons learned and inform relevant interventions for future pandemic responses.

## Results

### Socio-demographic and professional characteristics of participants

Table S1 shows the distribution of the study HCPs according to their sociodemographic and work characteristics. Three hundred and sixty-two HCPs participated in the study and were equally recruited from Egypt (181) and Sudan (181) with mean age (standard deviation [SD]) of the whole sample of 31.84 ± 8.32 years or 37.0 ± 7.55 years for Egypt, and 26.67 ± 5.31 years for Sudan. More than half of the study HCPs were females (60%), 50.3% were single and 55% completed a bachelor’s degree. Physicians constituted 58.3% of the professions with 32.7%, and 23.2% from the emergency and ICU departments respectively. Nurses constituted 28.2% of the professions with more than half of them working in the ICU department (58.8%) and 37.3% working in the emergency department, and of the total study pharmacists; 80.8% worked in the hospital pharmacy. Most HCPs (43.6%) had only one day of the week off. The mean working hours per week were 41 ± 19.22 hr/week for the whole sample.

### Burnout frequency

The majority of healthcare providers had high levels of work disengagement (75.4%) and emotional exhaustion (98.6%). Burnout syndrome was shown in 75% of the healthcare providers with 77% occurrence among the Egyptian HCPs and 71% occurrence among the Sudanese HCPs (Table 1).

**Table 1:** Distribution of healthcare professionals working at COVID-19 isolation facilities in Egypt and Sudan according to levels of burnout domains.

	N (%)	Nationality	
		Egypt (n = 181); n (%)	Sudan (n = 181); n (%)
Disengagement			
Low	89 (24.6%)	38 (21.0%)	51 (28.2%)
High	273 (75.4%)	143 (79.0%)	130 (71.8%)
Exhaustion			
Low	5 (1.4%)	3 (1.7%)	2 (1.1%)
High	357 (98.6%)	178 (98.3%)	179 (98.9%)
Overall burnout			
Non-burnout	4 (1.1%)	3 (1.7%)	1 (0.6%)
Disengaged	1 (0.3%)	0 (0.0%)	1 (0.6%)
Exhausted	85 (23.5%)	35 (19.3%)	50 (27.6%)
Burnout	272 (75.1%)	143 (79.0%)	129 (71.3%)

Table 2 shows the mean scores of the two domains of burnout. The mean score of disengagement for all HCPs of both countries was 2.56 ± 0.53, for Egypt (2.65 ± 0.57), and Sudan (2.47 ± 0.47). The mean score of emotional exhaustion for all HCPs of both countries was 2.73 ± 0.29, for Egypt (2.76 ± 0.28), and for Sudan (2.70 ± 0.29). The mean scores of overall burnout for all HCPs from both countries were 2.64 ± 0.36, for Egypt (2.70 ± 0.38), and 2.58 ± 0.34 for Sudan.

**Table 2:** Mean scores of burnout syndrome among healthcare professionals working at COVID-19 isolation facilities in Egypt and Sudan.

The mean score of burnout domains	Total (n = 362)		Nationality			
			Egypt (n = 181)		Sudan (n = 181)	
	Mean ± SD	Median	Mean ± SD	Median	Mean ± SD	Median
Mean score of disengagement	2.56 ± 0.53	2.63	2.65 ± 0.57	2.75	2.47 ± 0.47	2.50
Mean score of exhaustion	2.73 ± 0.29	2.75	2.76 ± 0.28	2.75	2.70 ± 0.29	2.75
Mean score of the overall burnout	2.64 ± 0.36	2.63	2.70 ± 0.38	2.75	2.58 ± 0.34	2.56

### Factors associated with burnout syndrome

Among Egyptian HCPs, the educational qualification (p = 0.001) and profession (p ≤ 0.001) showed a statistically significant relationship with the work disengagement mean scores. Age, profession, and working hours per week showed a significant relation with emotional exhaustion mean scores (p = 0.004, 0.020, 0.046 respectively). While the overall burnout mean scores were statistically significantly related to age, educational qualification, and profession (p = 0.015, 0.002, < 0.001 respectively) (Table S2).

For Sudanese HCPs, age showed a statistically significant relationship with the mean scores of work disengagement, emotional exhaustion, and overall burnout (*p* = 0.009, < 0.001, < 0.001 respectively) as well as the number of days off (*p* ≤ 0.001). There was a significant relationship between educational qualifications and work disengagement (*p* = 0.003) and overall burnout mean scores (*p* = 0.003). While the mean scores for emotional exhaustion differed significantly according to the profession (*p* ≤ 0.001) (Table S3).

### Predictors of burnout syndrome

The analysis of factors affecting burnout was contextualized to each different setting using univariate and multivariate regression models. Table 3 shows that, among Egyptian HCPs, the independent parameters from the sociodemographic and work-related characteristics that affected the burnout syndrome from the univariate regression analysis with statistical significance were educational qualifications (β = 1.231, *p* = 0.002), certain professions as doctors (β = 4.567, *p* ≤ 0.001), nurses (β = 4.977, *p* = 0.001), radiology technicians (β = 13.289, *p* = 0.029) and the working hours per week (β = 0.046, *p* = 0.029). However, after adjusting for all other independent variables the multivariate analysis showed that working hours were the only significant predictor for burnout syndrome among Egyptian HCPs where one hour increase in working hours per week is associated with a 0.04 increase in levels of burnout domains (β = 0.044, *p* = 0.031).

**Table 3:** Regression analysis for the parameters affecting burnout syndrome among frontline healthcare professionals in Egypt.

Variables	Univariate		Multivariate	
	p	β (95% CI)	p	β (95% CI)
Age (years)	0.682	− 0.025 (− 0.144 to 0.094)		
Sex	0.582	0.525 (− 1.357 to 2.407)		
Marital Status	0.660	0.456 (− 1.589 to 2.502)		
Qualification	0.002*	1.231 (0.444–2.018)	0.235	0.520 (− 0.342 to 1.383)
Profession				
Doctors	< 0.001*	4.567 (2.532–6.602)	0.404	1.390 (− 1.889 to 4.669)
Pharmacists	0.858	− 0.315 (− 3.790 to 3.160)		
Nurses	0.001*	− 4.977 (− 7.964 to − 1.990)	0.088	− 3.620 (− 7.788 to 0.548)
Lab technician	0.098	− 4.188 (− 9.161 to 0.786)		
Radiology technician	0.029*	− 13.289 (− 25.234 to − 1.344)	0.335	− 2.706 (− 8.226 to 2.814)
Days off	0.060	1.120 (− 0.048 to 2.288)		
Working hours/week	0.029*	0.046 (0.005–0.088)	0.031*	0.044 (0.004–0.085)

R^2^ = 0.147, F = 5.007*, p < 0.001* β: Unstandardized Coefficients C.I: Confidence interval *: significant at p ≤ 0.05.

For Sudanese HCPs, the independent parameters from the univariate regression analysis that affected burnout syndrome with statistical significance were the age (β = 0.277, *p* ≤ 0.001), educational qualification (β = 1.162, *p* = 0.038), professions such as pharmacists (*p* = 0.003), nurses (β = 2.038, *p* = 0.011), radiology technicians (β = 6.820, p = 0.030) and the number of days off (β = 2.112, p ≤ 0.001). After adjustment for all possible confounders, age as a predictor and the number of days off as a protective factor were the only significant parameters affecting burnout syndrome in the multivariate analysis (β = 0.230, -2.155, p = 0.022, < 0.001 respectively) where one year increase in age is associated with 0.23 increase in the levels of burnout domains (disengagement and emotional exhaustion). On the other hand, an increase of one day off is associated with a 2.1 decrease in the levels of burnout domains (Table 4).

**Table 4:** Regression analysis for the parameters affecting burnout syndrome among frontline healthcare professionals in Sudan.

Variables	Univariate		Multivariate	
	p	β (95% CI)	p	β (95% CI)
Age (years)	< 0.001*	0.277 (0.132–0.421)	0.022*	0.230 (0.034–0.426)
Sex	0.363	− 0.736 (− 2.328 to 0.857)		
Marital status	0.861	− 0.175 (− 2.146 to 1.795)		
Qualification	0.038*	1.162 (0.064–2.259)	0.410	0.484 (− 0.673 to 1.641)
Profession				
Doctors	0.396	0.700 (− 0.924 to 2.324)		
Pharmacists	0.003*	4.657 (1.657–7.657)	0.076	2.653 (− 0.280 to 5.585)
Nurses	0.011*	− 2.038 (− 3.602 to − 0.475)	0.473	− 0.584 (− 2.185 to 1.017)
Lab technician	0.475	− 1.493 (− 5.609 to 2.622)		
Radiology technician	0.030*	6.820 (0.678–12.962)	0.552	2.199 (− 5.091 to 9.489)
Days off	< 0.001*	− 2.112 (− 3.025 to − 1.200)	< 0.001*	− 2.155 (− 3.049 to − 1.262)
Working hours/week	0.918	0.002 (− 0.045 to 0.050)		

R^2^ = 0.225, F = 8.402*, p < 0.001*. β: Unstandardized Coefficients C.I: Confidence interval.

*Statistically significant at p ≤ 0.05.

### Parameters affecting burnout syndrome among frontline healthcare professionals

The independent parameters from the univariate regression analysis that affected burnout syndrome with statistical significance were the country (β = − 4.006, *p* = 0.002), the age (β = 0.246, *p* = 0.001), educational qualification (β = 2.856, *p* ≤ 0.001), professions such as doctors (β = 5.713, *p* ≤ 0.001) and nurses (β = − 6.652, *p* ≤ 0.001), and the number of days off (β = − 1.734, p ≤ 0.028). After adjustment for all possible confounders, the educational qualification and the number of days off were the only significant parameters affecting burnout syndrome in the multivariate analysis (β = 2.058, − 1.926, p = 0.006, 0.014 respectively) where one year increase in age is associated with 2.05 increase in the levels of burnout domains. On the other hand, an increase of one day off is associated with a 1.9 decrease in the levels of burnout domains (Table S4).

## Discussion

Since the emergence of the COVID-19 pandemic, healthcare workers across the world played a pivotal role in treating COVID-19 patients and being on the frontline directly exposed to  the patients was with a potentially significant burden to their health and well-being^17^. Compared to normal circumstances, there is a significant increase in pandemic-related burnout^18^, and the potential mental health impact of COVID-19 on frontline HCPs should be considered. The current study aimed to assess the extent of burnout among HCPs working in COVID-19 isolation facilities in Egypt and Sudan and to determine the associated sociodemographic and work characteristics.

In accordance with our hypothesis that there is a high prevalence of burnout syndrome among front-line HCPs during the COVID-19 pandemic in both African countries. The results of the current study revealed a high percentage of burnout in both countries, which was 77% among the Egyptian HCPs and 71% among the Sudanese HCPs. These results were less than COVID-19-related burnout which was 89.1% among nurses working in a university hospital in Italy^19^, which was conducted during the first wave of the COVID-19 pandemic in contrast to the current study which was conducted during the third wave of the COVID-19 pandemic. Moreover, the frequency of burnout in the current study is less than the reported levels among Egyptian ICU workers which was 87.4%^20^. On the other hand, the results of the current study were similar to the results of other studies which showed 63% burnout prevalence in the UK, 71% in Poland, 68% in Singapore^3^, and 73% in Egypt^21^. Current study findings were more than the reported burnout prevalence among medical residents in Sao Paulo, Brazil which was 49%^22^, and more than the reported prevalence of 51% among HCPs during an intercontinental survey from 33 countries during the COVID-19 pandemic but the study was based on measuring only the exhaustion core domain of burnout^14^.

Differences in the reported levels of burnout syndrome in the published literature during COVID-19 can be due to the situation of the pandemic at the time of data collection in each country, the method which was used for assessing burnout which was commonly the Maslach Burnout Inventory, and some studies used Copenhagen Inventory and others^23^, or the nature of the health system in different countries^20^. Other reasons that may explain the high frequency of burnout in the current study are that is based on the assessment of burnout syndrome among the HCPs working in the isolation facilities; the increased workload, increased psychological distress, the burden from the direct contact with COVID-19 patients, and the stress of dealing with a newly emergent disease in addition to the fear of self-infection or infecting their relatives collectively can cause increased burnout levels in comparison to HCPs working in non-isolation health facilities^24–26^. In addition to that, working among isolated patients is likely to experience multiple mental health problems, and many HCPs working at isolation facilities are withdrawn or suggested self-isolation after working on COVID-19 cases^27^. Although this explanation was contradictory to other studies which stated that non-frontline HCPS show a higher level of burnout and stress than frontline HCPS, explained by the isolation facilities are better organized and there is a sense of more control while the fear of being exposed when the protocols and organizations do not seem well established is the situation in non-isolation facilities^28^.

A study conducted among Egyptian physicians working during the COVID-19 pandemic in non-isolation health facilities revealed a 36% burnout prevalence less than the current study using a different scale^13^. Reasons for this discrepancy could be clarified by a study that was conducted among Egyptian nurses working in one of the COVID-19 triage hospitals which showed that three-quarters of nurses (75.2%) had high occupational stress levels. The highest priority stressors that were reported were dealing with death and dying, having personal demands and fears, employing strict biosecurity measures, and, the COVID-19 stigma^29^. Finally, the presence of workload as frontline healthcare facilities are faced with the early rush of COVID-19 cases being the specialized tertiary care facilities provided with all methods for the diagnosis and management of suspected and confirmed COVID-19 cases^30^.

The frequency of burnout among Sudanese HCPs participated in the present study was greater than that among Sudanese HCPs working at primary health care centers in Wad Madani Al-Kubra in Sudan during the COVID-19 pandemic which was 45% using Maslach Burnout Inventory and conducted in a different setting^31^. In addition, a study among resident physicians in Sudan during the COVID-19 pandemic reported high rates of burnout among this population^32^. Another study concluded that more than half of HCPs working in Khartoum state hospitals showed high levels of stress^33^. Due to the fragile health system in Sudan, political instability, and economic meltdown; medical personnel has to work under high pressure in a very limited infrastructure, and as a sequel working under stress and pressure can be the leading factors that make HCPs more prone to burnout^33,34^.

In the current study, the majority of HCPs of both countries had high levels of disengagement (75.4%) and emotional exhaustion (98.6%) which is higher than the findings among the Italian nurses who showed high levels of exhaustion (76%) and disengagement (52%)^19^. Our results were close to the results of healthcare workers in Singapore which revealed 79.7% disengagement and 75.3% exhaustion with mean OLBI scores of 2.38 and 2.50 for disengagement and exhaustion respectively^4^ close to the mean scores of the current study which were 2.56 and 2.73 for disengagement and exhaustion respectively. The mean score of burnout of all participants of the present study was 2.64 ± 0.36 consistent with frontline nurses working in COVID-19 wards in Iran hospitals where the mean score of burnout was 2.61 ± 0.27^35^.

In accordance with the second hypothesis in this research, there are certain socio demographic and specific professional characteristics that predict the occurrence of burnout among frontline HCPs. Age from 20 to 40 years was significantly associated with higher levels of emotional exhaustion among Egyptian study participants similar to findings of other studies^13,20,26,36,37^, and this was explained by a study conducted by Dimitriu et al. that younger physicians always more work loaded and faced continuously with unpredictable changes in duty schedules and cancelation of vacations. Others explained that older HCPs may have better knowledge in comparison to younger ones in coping with burnout^26^. While other studies reasoned that young HCPs are more exposed to social media which is full of information about the pandemic, some necessary and some unnecessary disturbing news that can be stressful^38^. But this was not the condition among the study Sudanese participants as being older than 40 years was significantly associated with more levels of emotional exhaustion and supporting these results was that older practitioners fear more exposure risk to infection and occurrence of complications especially if they have underlying diseases^39^.

Educational qualification of the study HCPs was significantly associated with the levels of burnout domains similar to findings of other studies^21^. The profession was significantly associated with levels of burnout and levels of emotional exhaustion among the current study participants similar to findings of other studies^40^, and the higher levels of burnout were among the study Egyptian physicians and pharmacists as well as Sudanese radiology technicians and pharmacists.

Increasing working hours per week was associated with higher levels of emotional exhaustion among Egyptian HCPs similar to that reported in Galván^41^ and Hathout^30^ studies and to the levels of patient-related burnout reported among emergency physicians working > 40 h using the Copenhagen tool^10^. While the increasing hours were found not significantly different in another study^20^. It was found that the reasons behind the psychological consequences caused by the pandemic to HCPs were working more hours than usual can be exceeded to making double shifts. This may lead to a disturbance of the circadian rhythm and the sleep–wake cycle of HCPs, job stress can lead to malpractices and unsatisfactory job performance. Long working hours also cause more exposure to patients^26,30^. Long working hours may have an impact on the social life and family of women HCPs and may result in the development of fear and guilt toward their families^13^. Resting time is always needed to guarantee personal wellness and proper job performance^42^, as inadequate leisure time appears to be one of the major stressors and is associated with a high level of burnout^20^.

Findings from the present study indicated that the significant parameter affecting burnout syndrome among Egyptian health professionals was working hours per week similar to results reported in another study^43^. The unsuitable working hours have greatly decreased provider satisfaction among this population^44^, with the additional workload imposed by the COVID-19 pandemic, which might have contributed to further decreased job satisfaction and increased the possibility of burnout development among participants. Following this finding, a study among HCPs amid COVID-19 has found that working hours are significantly associated with burnout^45^. Working hours were one of the independent significant factors associated with stress in other studies^46^. While amongst Sudanese HCPs, participants’ age was found to be positively associated with burnout and the number of days off as a protective factor, a similar study among nurses concluded that age may be associated with a reduced sense of achievement at work.^47^ Additionally, research on burnout showed that age followed a non-linear relationship with emotional exhaustion and total burnout^48^. In support of our findings, a study revealed that the number of days off is a potentially protective factor against burnout^49^.

Based on the study findings, it is important to address both organizational and individual contributors to burnout in both countries to create a healthy and sustainable work environment. This can include strategies such as providing adequate resources, promoting work-life balance, recognizing and rewarding employees' efforts, and offering mental health resources and support. Additionally, individuals can take steps to manage their own stress and prioritize self-care, such as setting boundaries, practicing mindfulness, and seeking support when needed.

This study encountered several limitations. The study followed a cross-sectional study design; hence, it’s difficult to establish the causal association between the study variables and burnout syndrome. The baseline level of burnout before the pandemic was not assessed, comparing the changes in the level of burnout would have certainly added to this study. Moreover, the use of non-probability sampling in recruiting participants in the cross-sectional survey might have affected the representativeness of our sample and limited the generalization of study findings. Finally, the use of an online survey introduced the risk of selection bias, favoring HCPs who had access to an internet connection. Despite these limitations, the study results are not inconsistent with published literature and were able to shed light on COVID-19-related mental health issues among this critical population.

## Conclusions

The study revealed a high frequency of burnout syndrome among HCPs working at the COVID-19 isolation facilities in Egypt and Sudan. Appropriate action should be taken to preserve mental health status through the establishment of effective and efficient coping strategies.

## Methods

### Study design and sampling

Participants were recruited through a non-probability convenience sampling and HCP was asked to invite his colleagues to participate. Assuming the expected population standard deviation to be 10, with 95% confidence and a precision of 1.5, the minimum required sample size was 174 participants from each country. The total number of participants collected from both countries was 362.

### Participant selection

Since the study was conducted amidst the COVID-19 pandemic within a strict isolation policy that calls for reducing face-to-face contact, a cross-sectional internet-based survey was conducted for three months starting from May 2021 till the end of July 2021. The online survey form was created using Google Forms and a link to the electronic questionnaire was distributed to respondents across social media platforms; Facebook and WhatsApp which are exclusive for HCPs working at COVID-19 isolation facilities in Egypt and Sudan, as used in previous surveys of HCPs during the pandemic^50^. Admins of these groups were contacted and asked to circulate and post the questionnaire on their groups and among their HCPs. Physicians, nurses, pharmacists, and paramedical technicians of different specialties working at COVID-19 isolation facilities in Egypt and Sudan at the time of data collection were invited to participate in the study and to submit and distribute the online questionnaire. The reception of the filled questionnaires was stopped when the target sample size had been achieved.

### Survey tool and data collection

Anonymous Google form, a self-administered questionnaire was used for data collection. The required data related to sociodemographic and professional characteristics (country, age, sex, marital status, educational qualification, profession and specialty, days off, and working hours/week) were collected. Occupational burnout was estimated using Oldenburg Burnout Inventory (OLBI). The OLBI is a validated tool for the investigation of burnout, it consists of 16 items; eight of the questions are related to emotional exhaustion, and eight are about job disengagement. Items comprise both positively and negatively phrased questions recorded on a four-point Likert scale ranging from 1 to 4 (Strongly agree–Strongly disagree) with four points for the highest burnout response and one point for the lowest^51^. Participants were considered to be "at high risk of burnout" if they met the thresholds of 2.1 for the exhaustion subscales and 2.25 for the disengagement subscales^52,53^. OLBI outcomes were defined by four subscale scores that are calculated as the mean of the item scores for each subscale^54–56^ and categorized as follows.

**Table:** 

*Burnout* High exhaustion score ≥ 2.25 and high disengagement score ≥ 2.1
*Exhausted* High exhaustion score ≥ 2.25 and low disengagement score < 2.1
*Disengaged* High disengagement score ≥ 2.1 and low exhaustion score < 2.25
*No burnout* Low exhaustion score < 2.25 and low disengagement score < 2.1

The OLBI items were translated into Arabic by the researchers, the questionnaire was piloted on 30 HCPs to examine understandability and face validity. The Internal consistency of OLBI subscales was measured using Cronbach alpha. For the 8 items of disengagement, the value of Cronbach's alpha was 0.7, and for the 8 items of exhaustion was 0.85 and for the overall burnout of the total 16 items, Cronbach’s alpha was 0.88 which is considered acceptable^57^. The final questionnaire was distributed virtually through social media platforms that are exclusive for HCPs working at COVID-19 isolation hospitals in Egypt, and Sudan.

### Data analysis

Data was fed to the computer and analyzed using IBM SPSS software package version 20.0. (Armonk, NY: IBM Corp). The Kolmogorov- Smirnov test was used to verify the normality of the distribution of variables. Mean with standard deviation, and frequencies with percent were used to describe the numerical and categorical data respectively. The student’s t-test was used to compare the means between two groups for normally distributed quantitative variables. ANOVA was used for comparing the means between more than two groups. Univariate and multivariate regression analysis was conducted for identifying the independent sociodemographic and work parameters affecting burnout syndrome. All results were considered statistically significant when the significant probability was less than 5% (*p* < *0.05*).

### Ethical considerations

This study was performed in accordance with the ethical standards as laid down in the 1964 Declaration of Helsinki and its later amendments or comparable ethical standards. This study was approved by the Ethics and Technical Committee of the High Institute of Public Health, Alexandria University in May 2021. The purpose of the research was started at the beginning of the questionnaire and informed consent was taken from all participants. Participants were able to withdraw from the survey any time before its completion by not pressing submission.

## Supplementary Information

Supplementary Tables.

## Data Availability

The data supporting the current study findings are available from the corresponding author upon reasonable request.
