# Supplementary material for: High levels of burnout among health professionals treating COVID-19 patients in two Nile basin countries with limited resources
Source: Sci Rep. 2023 Apr 20;13:6455. doi: 10.1038/s41598-023-33399-2 (PMC10116483; doi:10.1038/s41598-023-33399-2)
Supplement: Supplementary file 1 — Supplementary Tables. [file 41598_2023_33399_MOESM1_ESM.pdf]

Supplementary information

Table S1. Characteristics of study participants (n= 362).

Socio-demographic characteristics		Total n(%)	Country	
			Egypt (181) n (%)	Sudan (181) n (%)
**Age (years)**				
20 - <40		293 (80.9%)	118 (65.2%)	175 (96.7%)
40 - <60+		69 (19.1%)	63 (34.8%)	6 (3.3%)
Mean ± SD.		31.84 ± 8.32	37.0 ± 7.55	26.67 ± 5.31
**Sex**				
Male		145 (40.1%)	63 (34.8%)	82 (45.3%)
Female		217 (59.9%)	118 (65.2%)	99 (54.7%)
**Marital Status**				
Single		182 (50.3%)	40 (22.1%)	142 (78.5%)
Married		171 (47.2%)	134 (74.0%)	37 (20.4%)
Divorced/widowed		9 (2.5%)	7 (3.9%)	2 (1.1%)
**Educational Qualification**				
Diploma		17 (4.7%)	12 (6.6%)	5 (2.8%)
Bachelor		199 (55.0%)	45 (24.9%)	154 (85.1%)
Postgraduate Diploma		38 (10.5%)	34 (18.8%)	4 (2.2%)
Master's/Fellowship		88 (24.3%)	74 (40.9%)	14 (7.7%)
PhD		20 (5.5%)	16 (8.8%)	4 (2.2%)
**Profession**	**Department**			
**Doctors 211 (58.3%)**	ICU	49 (23.2%)	33 (23.6%)	16 (22.5%)
	Surgery	15 (7.1%)	9 (6.4%)	6 (8.5%)
	Internal medicine	40 (19.0%)	25 (17.9%)	15 (21.1%)
	Emergency	69 (32.7%)	39 (27.9%)	30 (42.3%)
	Others	38 (18.0%)	34 (24.3%)	4 (5.6%)
**Pharmacists 26 (7.2%)**	Clinical Pharmacy	5 (19.2%)	5 (38.5%)	0 (0.0%)
	Hospital Pharmacy	21 (80.8%)	8 (61.5%)	13 (100.0%)
**Nurses 102 (28.2%)**	ICU	60 (58.8%)	12 (70.6%)	48 (56.5%)
	Emergency	38 (37.3%)	1 (5.9%)	37 (43.5%)
	Others	4 (3.9%)	4 (23.5%)	0 (0.0%)
**Lab technician**		13 (3.6%)	6 (3.3%)	7 (3.9%)
**Radiology technician**		4 (1.1%)	1 (0.6%)	3 (1.7%)
**Others**		6 (1.7%)	4 (2.2%)	2 (1.1%)
**Days off**				
1 day		158 (43.6%)	94 (51.9%)	64 (35.4%)
2 days		113 (31.2%)	55 (30.4%)	58 (32.0%)
3 days or more		91 (25.1%)	32 (17.7%)	59 (32.6%)
**Working hours /Week**				
<36		106 (29.3%)	53 (29.3%)	53 (29.3%)
36-<42		122 (33.7%)	63 (34.8%)	59 (32.6%)
≥42		134 (37.0%)	65 (35.9%)	69 (38.1%)
Mean ± SD.		41.08 ± 19.22	41.03 ± 21.42	41.13 ± 16.80

Table S2. Relation between burnout syndrome and the sociodemographic and work characteristics of Egyptian participants.

Socio-demographic characteristics	N	Disengagement	Exhaustion	Overall burnout
		Mean ± SD.	Mean ± SD.	Mean ± SD.
**Age (years)**				
20 - <40	**118**	21.61 ± 4.68	22.41 ± 2.02	44.02 ± 5.91
40 - <60+	**63**	20.32 ± 4.33	21.40 ± 2.52	41.71 ± 6.22
**t(p)**		**1.817 (0.071)**	**2.933*(0.004*)**	**2.453*(0.015*)**
**Sex**				
Male	**63**	20.84 ± 5.12	22.03 ± 2.43	42.87 ± 6.95
Female	**118**	21.33 ± 4.29	22.07 ± 2.16	43.40 ± 5.62
**t(p)**		**0.682(0.496)**	**0.102(0.919)**	**0.551(0.582)**
**Marital Status**				
Single	**40**	21.47 ± 4.86	22.0 ± 2.25	43.47 ± 6.13
Married	**134**	21.01 ± 4.52	22.09 ± 2.26	43.10 ± 6.12
Divorced/widowed	**7**	22.29 ± 4.79	21.71 ± 2.50	44.0 ± 6.35
**F(p)**		**0.376(0.687)**	**0.107(0.899)**	**0.118(0.889)**
**Qualification**				
Diploma	**12**	16.42 ± 5.57	20.75 ± 3.05	37.17 ± 7.64
Bachelor	**45**	20.62 ± 4.82	22.18 ± 2.07	42.80 ± 6.02
Postgraduate Diploma	**34**	20.94 ± 3.84	21.74 ± 2.44	42.68 ± 5.64
Master's/Fellowship	**74**	22.31 ± 4.17	22.36 ± 2.08	44.68 ± 5.53
PhD	**16**	21.38 ± 4.40	21.94 ± 2.29	43.31 ± 6.11
**F(p)**		**4.951*(0.001*)**	**1.593 (0.178)**	**4.445*(0.002*)**
**Profession**				
Doctors	**140**	22.05 ± 4.17	22.20 ± 2.14	44.25 ± 5.67
Pharmacists	**13**	20.15 ± 4.67	22.77 ± 2.45	42.92 ± 5.82
Nurses	**17**	17.29 ± 4.10	21.41 ± 2.27	38.71 ± 5.01
Lab technician	**6**	17.83 ± 5.00	21.33 ± 1.51	39.17 ± 5.78
Radiology technician	**1**	13.0	17.0	30.0
Others	**4**	16.75 ± 6.50	19.75 ± 3.86	36.50 ± 10.08
**F(p)**		**6.438*(<0.001*)**	**2.750*(0.020*)**	**5.784*(<0.001*)**
**Days off**				
1 day	**94**	20.53 ± 4.88	21.86 ± 2.52	42.39 ± 6.57
2 days	**55**	21.58 ± 3.78	22.31 ± 1.84	43.89 ± 5.00
3 days or more	**32**	22.28 ± 4.80	22.19 ± 2.05	44.47 ± 6.21
**F(p)**		**2.093 (0.126)**	**0.749(0.474)**	**`1.884(0.155)**
**Working hours /Week**				
<36	**53**	20.34 ± 4.19	21.51 ± 2.14	41.85 ± 5.53
36-<42	**63**	21.33 ± 4.76	22.02 ± 2.42	43.35 ± 6.43
≥42	**65**	21.66 ± 4.71	22.54 ± 2.09	44.20 ± 6.10
**F(p)**		**1.284(0.279)**	**3.133*(0.046*)**	**2.221 (0.112)**

t: Student t-test F: ANOVA test *: Statistically significant at p ≤ 0.05

Table S3. Relation between burnout syndrome and the sociodemographic and work characteristics of Sudanese participants.

Socio-demographic characteristics	N	Disengagement	Exhaustion	Overall burnout
		Mean ± SD.	Mean ± SD.	Mean ± SD.
**Age (years)**				
20 - <40	**175**	19.59 ± 3.70	21.43 ± 2.19	41.02 ± 5.20
40 - <60+	**6**	23.67 ± 4.08	25.50 ± 1.76	49.17 ± 5.71
**t(p)**		**2.649*(0.009*)**	**4.498*(<0.001*)**	**3.761*(<0.001*)**
**Sex**				
Male	**82**	19.91 ± 3.54	21.78 ± 2.43	41.70 ± 5.09
Female	**99**	19.57 ± 3.96	21.39 ± 2.16	40.96 ± 5.65
**t(p)**		**0.619 (0.537)**	**1.131 (0.260)**	**0.911 (0.363)**
**Marital Status**				
Single	**142**	19.65 ± 3.76	21.59 ± 2.17	41.25 ± 5.30
Married	**37**	19.92 ± 3.93	21.51 ± 2.68	41.43 ± 5.93
Divorced/widowed	**2**	21.0 ± 0.0	21.0 ± 4.24	42.0 ± 4.24
**F(p)**		**0.186 (0.830)**	**0.079 (0.924)**	**0.034 (0.966)**
**Qualification**				
Diploma	**5**	22.0 ± 5.00	22.00 ± 2.45	44.0 ± 6.48
Bachelor	**154**	19.47 ± 3.55	21.47 ± 2.18	40.94 ± 5.08
Postgraduate Diploma	**4**	15.50 ± 4.93	19.50 ± 2.38	35.0 ± 6.63
Master's/Fellowship	**14**	22.43 ± 4.05	22.71 ± 2.67	45.14 ± 6.06
PhD	**4**	21.50 ± 2.65	23.00 ± 3.56	44.50 ± 4.43
**F(p)**		**4.190* (0.003*)**	**2.263 (0.064)**	**4.257* (0.003*)**
**Profession**				
Doctors	**71**	19.97 ± 3.47	21.75 ± 2.48	41.72 ± 5.07
Pharmacists	**13**	22.08 ± 5.01	23.54 ± 2.63	45.62 ± 6.69
Nurses	**85**	19.21 ± 3.74	21.0 ± 1.88	40.21 ± 5.16
Lab technician	**7**	18.14 ± 3.58	21.71 ± 1.38	39.86 ± 4.81
Radiology technician	**3**	23.0 ± 1.73	25.0 ± 1.73	48.0 ± 3.46
Others	**2**	18.0 ± 1.41	21.0 ± 1.41	39.00 ± 0.0
**F(p)**		**2.246 (0.052)**	**4.915*(<0.001*)**	**3.804*(0.003*)**
**Days off**				
1 day	**64**	20.56 ± 3.45	22.38 ± 2.03	42.94 ± 4.68
2 days	**58**	20.48 ± 3.54	21.66 ± 2.52	42.14 ± 5.37
3 days or more	**59**	18.07 ± 3.85	20.61 ± 1.97	38.68 ± 5.27
**F(p)**		**9.224*(<0.001*)**	**10.100*(<0.001*)**	**11.864*(<0.001*)**
**Working hours /Week**				
<36	**53**	19.28 ± 4.40	21.23 ± 2.59	40.51 ± 6.28
36-<42	**59**	19.36 ± 3.24	21.39 ± 2.03	40.75 ± 4.58
≥42	**69**	20.38 ± 3.63	21.99 ± 2.22	42.36 ± 5.22
**F(p)**		**1.691 (0.187)**	**1.934 (0.148)**	**2.242 (0.109)**

t: Student t-test F: ANOVA test *: Statistically significant at $p \le 0.05$

Table S4. Linear regression analysis for the parameters affecting burnout syndrome among frontline healthcare professionals (N= 362).

Variables	Univariate		Multivariate	
	p	β (95%C.I)	p	β (95%C.I)
**Country (Egypt)**	0.002*	–4.006 (-6.487– -1.524)	0.832	0.349 (-3.591–2.892)
**Age (years)**	0.001*	0.246 (0.096– 0.395)	0.830	-0.022 (-0.221–0.178)
**Sex**	0.900	0.164 (-2.403– 2.731)		
**Marital Status**	0.081	2.038 (-0.252– 4.328)		
**Qualification**	<0.001*	2.857 (1.704– 4.009)	0.006*	2.058 (0.598–3.518)
**Profession**				
**Doctors**	<0.001*	5.713(3.232– 8.195)	0.333	1.847 (-1.897–5.591)
**Pharmacists**	0.067	4.523 (-0.327– 9.373)		
**Nurses**	<0.001*	-6.652 (-9.362–-3.942)	0.115	-3.284(-7.375–0.808)
**Lab technician**	0.087	-5.868 (-12.602–0.865)		
**Radiology technician**	0.668	2.625 (-9.407–14.656)		
**Days off**	0.028*	-1.734 (-3.280– -0.189)	0.014*	-1.926 (-3.466–-0.385)
**Working hours /Week**	0.066	0.061 (-0.004– 0.126)		
